# β2 Integrin-Mediated Susceptibility to *Paracoccidioides brasiliensis* Experimental Infection in Mice

**DOI:** 10.3389/fcimb.2021.622899

**Published:** 2021-03-16

**Authors:** Stephan Alberto Machado de Oliveira, Janayna Nunes Reis, Elisa Catão, Andre Correa Amaral, Ana Camila Oliveira Souza, Alice Melo Ribeiro, Lúcia Helena Faccioli, Fabiana Pirani Carneiro, Clara Luna Freitas Marina, Pedro Henrique Bürgel, Larissa Fernandes, Aldo Henrique Tavares, Anamelia Lorenzetti Bocca

**Affiliations:** ^1^ Molecular Pathology Graduation Course, Faculty of Medicine, University of Brasilia, Brasilia, Brazil; ^2^ Department of Cell Biology, Institute of Biological Sciences, University of Brasilia, Brasilia, Brazil; ^3^ Institute of Tropical Pathology and Public Health, Federal University of Goiás, Goiania, Brazil; ^4^ Faculty of Pharmaceutical Sciences of Ribeirão Preto, University of São Paulo, Ribeirão Preto, Brazil; ^5^ Area of Pathology, Faculty of Medicine, University of Brasilia, Brasilia, Brazil; ^6^ Molecular Biology Graduation Course, Institute of Biological Sciences, University of Brasilia, Brasilia, Brazil; ^7^ Faculty of Ceilândia, University of Brasília, Brasília, Brazil

**Keywords:** CD18^low^ mice, nitric oxide, β2 integrin, *Paracoccoidioides brasiliensis*, susceptibility

## Abstract

The earliest interaction between macrophages and *Paracoccidioides brasiliensis* is particularly important in paracoccidioidomycosis (PCM) progression, and surface proteins play a central role in this process. The present study investigated the contribution of β2 integrin in *P. brasiliensis*-macrophage interaction and PCM progression. We infected β2-low expression (CD18^low^) and wild type (WT) mice with *P. brasiliensis* 18. Disease progression was evaluated for fungal burden, lung granulomatous lesions, nitrate levels, and serum antibody production. Besides, the *in vitro* capacity of macrophages to internalize and kill fungal yeasts was investigated. Our results revealed that CD18^low^ mice infected with Pb18 survived during the time analyzed; their lungs showed fewer granulomas, a lower fungal load, lower levels of nitrate, and production of high levels of IgG1 in comparison to WT animals. Our results revealed that *in vitro* macrophages from CD18^low^ mice slowly internalized yeast cells, showing a lower fungal burden compared to WT cells. The migration capacity of macrophages was compromised and showed a higher intensity in the lysosome signal when compared with WT mice. Our data suggest that β2 integrins play an important role in fungal survival inside macrophages, and once phagocytosed, the macrophage may serve as a protective environment for *P. brasiliensis.*

## Introduction

*Paracoccidioides brasiliensis* (Pb) is a facultative intracellular fungus that causes paracoccidioidomycosis (PCM), a deep, chronic, and granulomatous disease prevalent in Latin America (Bocca et al., 2013). The disease manifests in multiple forms that range from benign and localized lesions to severe and disseminated infection, depending on the extent of the lowering of cellular immunity (Restrepo et al., 2008; Mendes et al., 2017). As described for other systemic mycoses, cellular immune response, mediated mainly by IFN-γ activated macrophages, is the host’s major defense mechanism against PCM (Bocca et al., 1999; Souto et al., 2000; Souto et al., 2003; Schimke et al., 2017). Activated macrophages show a fundamental role during all the disease outcomes, along with granuloma formation, to protect the host against the dissemination of the infection (Bocca et al., 1999; Souto et al., 2000; Pagliari et al., 2019). Granuloma formation relies on the secretion of cytokines such as IFN-γ and TNF-α, which confer resistance against Pb by macrophage activation, fungal contention, and nitric oxide (NO) production, resulting in the killing of the pathogen (Pagliari et al., 2019). Furthermore, IFN-γ modulates chemokines and chemokine receptors’ macrophage expression and the lung cellular infiltration pattern in mice experimentally infected with Pb (Souto et al., 2000). During PCM development, all antibody isotypes are increased in the highest amounts. They are reflected in the immune response’s polarization, since they are closely associated with Th1 and Th2 immune responses (Mamoni et al., 2002; Pinto et al., 2006; Tristão et al., 2013).

Although the host cellular immune response shows an essential role against infection, the interaction mechanisms involved in macrophage activation have not yet been thoroughly described. Due to the complexity of the interaction between the host and Pb, various studies have attempted to unveil the fungus’ innate host defense mechanisms (Calich et al., 2008; Pagliari et al., 2019). The interaction of host macrophages and Pb is mediated by cell surface receptors on the outer membrane of the macrophage, including mannose receptor, C-type lectin receptors (CTLR), such as dectin-1, Toll-like receptor 2 (TLR-2), TLR-4, surfactant protein, scavenger receptor, and complement receptor types 3 (CR3) and 4 (CR4) (Jimenez Mdel et al., 2006; Calich et al., 2008; Tan, 2012; Feriotti et al., 2013). Pb yeasts opsonized with fresh serum are more efficiently internalized than when opsonized with inactivated serum; therefore, CR3 shows particular importance in fungal internalization (Jimenez Mdel et al., 2006). The Pb yeast form can activate both the classical and alternative complement pathways *in vitro*, resulting in opsonization and phagocytosis by macrophages (Calich et al., 2008). CR3 is a receptor related to the fungal internalization by macrophages from both susceptible and resistant mice to fungus infection, while mannose receptors are associated only with phagocytes from resistant mice. This difference could influence the host’s susceptibility mechanisms during fungal infection (Jimenez Mdel et al., 2006). CR3 (CD11b/CD18) and CR4 (CD11c/CD18) share the beta subunit CD18, which is a heterodimer that belongs to the leukocyte β2-integrin family (Tan, 2012).

Integrin is a cell adhesion molecule that shows an important role in immunity, wound healing, and hemostasis (Tan, 2012). The β2 integrins comprise four members: LFA-1 (CD11a/CD18), Mac-1 or CR3 (CD11b/CD18), p150,95 or CR4 (CD11c/CD18), and αDβ2 (CD11d/CD18). CR3 and CR4 are mainly expressed in myeloid origin cells and mediate phagocytosis *via* iC3b-opsonized particles; they are also involved in monocytes’ adhesion to endothelial cells. Moreover, CR3 can mediate microorganism phagocytosis by recognizing the β 1-3 glucan component present on the cell wall of some fungi (Ross et al., 1987). The CR3 role in recognition and phagocytosis of different microbes has been described, including *Mycobacterium tuberculosis*, *Candida albicans*, *Francisella tularensis*, and *Cryptococcus neoformans* (Fukazawa and Kagaya, 1997; Velasco-Velasquez et al., 2003; Luo et al., 2006; Dai et al., 2013). Besides their phagocytosis role, during *Streptococcus pneumonie*, pulmonary infection, CR3 showed another important role in disease prevention: regulating neutrophil and T cell recruitment into the lung (Kadioglu et al., 2011). However, CR3 has also been related to a harmful role in the immune response to *Leishmania major* infection (Polando et al., 2013; Ricardo-Carter et al., 2013). The engagement of CR3 by various *Leishmania* ligands inhibits IL-12 and NO production by a mechanism independent of NFκB, MAPK, IRF, and ETS in an experimental model using CD11b-deficient mice (Ricardo-Carter et al., 2013). Furthermore, using the same experimental model, *Leishmania* opsonization with fresh serum influences phagosome trafficking and delays the maturation process (Polando et al., 2013).

Previous studies have linked an important role for CR3 in macrophage-Pb interaction and fungal phagocytosis. However, the CR3 role in the outcome of different experimental infection models is not a consensus. Here, we investigated the role of β2 integrins, low expression macrophages, during the *in vivo* and *ex-vivo P. brasiliensis* infection to better understand the importance of high internalization of fungal yeasts for fungal survival in macrophages.

## Materials and Methods

### Fungal Strain

The yeast form of a high virulent strain of *P. brasiliensis* (Pb18) was obtained from the fungal collection of the Laboratory of Applied Immunology’s fungal library. It was previously kindly provided by Dr. Peraçoli, from Unesp/Botucatu. It was maintained in mice, and to perform the experiments, fungal cells were recovered and grown in YPD culture medium at 36°C for five days. The yeast cells were then washed in phosphate-buffered saline (PBS) and adjusted to 1x10^7^ yeast/ml.

### Mice

Eight-week-old (n=24) C57BL/6 (WT) and homozygous *CD18low* mice of the C57BL/6 background were obtained from the animal facilities of the Pharmaceutical Science Faculty of Ribeirão Preto – University of São Paulo (FCFRP-USP), Brazil. The *CD18low* (B6.129S7-Itgb2^tm1bay^) mice were purchased at the Jackson Laboratory and serve as a model for the moderate form of human CD18 deficiency. Mice were placed in propylene cages in a controlled temperature room, fed with a standard diet, and given water *ad libitum*. The Animal Ethics Committee of the University of Brasilia approved all experiments using animal subjects (UnBDOC n°. 33798/2007).

*In vivo* experiments, mice were infected *via* intravenous (iv) route with 10^6^ yeast forms of Pb 18 to mimic a disseminated infection (n= 12 animal/group). At 15-, 30-, and 60-days post-infection, four animals per point were euthanized, and blood, lung, and spleen samples were aseptically collected for later analysis. For representative survival curves, we used the Kaplan-Meier estimator of an experimental intravenous infection carried out in WT and CD18^low^ mice with a suspension of 1×10^6^ Pb18, as previously described (Goel et al., 2010). Data were expressed as a percentage of live animals observed for 120 days (Granger et al., 1996). For *ex-vivo* analysis, mice were intraperitoneally inoculated with 3 ml of thioglycolate 3% and euthanized after four days for peritoneal macrophage collection.

### Fungal Burden Assay

Infection was assessed by counting the number of Colony Forming Units (CFUs) of *P. brasiliensis* recovered from infected mice’s lungs. Four animals from each group were euthanized by CO_2_ chamber at indicated time points, and the lungs were aseptically collected. One longitudinal section of each lung was weighed and macerated within sterilized PBS. One hundred µl from the homogenized lung tissues was plated into BHI agar supplemented with 4% horse serum; 5% *P. brasiliensis* 192 isolate yeast culture filtrated supernatant, and 40 mg/L of gentamicin (Gentamicin Sulfate, Schering-Plough, Rio de Janeiro, Brazil). Plates were incubated for seven days at 37°C, and CFUs were counted.

### Histopathologic and Histocytometry Analysis

Liver and lung fragments were removed from the two experimental groups and fixed in 10% phosphate-buffered formalin for 6 h, followed by 70% ethanol until embedding in paraffin. Several 5-µm sections were stained with H&E for light microscopic analysis. The diameters of the granulomatous lesions in the lung were quantified by histocytometry using an image analyzer (Image Pro-Plus Version 5.1.0.20 Copyright 1993-2004- Media Cybernectics, Inc.). and a computer and compared to the size of the fragment. The mean size of the lesions and the mean percentage of the lesioned area of the lung were also determined. The data were obtained by triplicate analysis of the sections performed by two observers.

### NO Production

The concentration of nitric oxide (NO) in the serum was determined by enzymatically reducing nitrate to nitrite with nitrate reductase, as previously described (Pina et al., 2008). The total amount of nitrite was then quantified by the Griess method. A microplate reader measured the absorbance at 540 nm.

### Lysosome Staining

To perform the staining of acid organelles, 1×10^5^ intraperitoneal macrophages were seeded in chamber slides and incubated at 37°C and 5% CO2. Next, the cells were co-incubated with *P. brasiliensis* (MOI 1:0.5) previously stained with Calcofluor (Sigma-Aldrich, St. Louis, MO, USA). After 24 h, extracellular fungi were washed with RPMI medium, and the cells were stained with Lysotracker^®^ Red DND-99 (Thermo Scientific, Waltham, MA, USA) (1:1,000) for 15 min at 37°C and directly used for microscopy using the Live Cell Imaging approach. Lysotracker Mean Fluorescent Intensity (MFI) was measured using ImageJ software.

### Antibody Isotypes Analysis

The specific IgG1 and IgG2a isotypes were measured in the serum by Enzyme-Linked ImmunoSorbent Assay (ELISA) (Sigma-Aldrich, St. Louis, MO, USA) as per the manufacturer’s instructions. Briefly, 96-well plates were coated overnight at 4°C with protein extract of the fungal cell wall (100 μl/well). The plates were blocked with mouse serum (1:100) for 2h at 37°C. The serum samples were added to the plates and incubated for 2 h at room temperature. After washing with PBS 0.05% Tween 20, peroxidase-labeled antibodies specific for mouse IgG1 or IgG2a isotypes were added (1:5,000), and plates were incubated for 2h at 37°C. Next, the plates were washed seven times with PBS 0.05% Tween 20 and incubated with H_2_O_2_ and o-phenylenediamine for the reaction. After the addition of 20 µl of H_2_SO_4_, 2N (stop solution), the reactions were read at 490 nm in an ELISA plate reader (BioRad, model 2550, Hercules, CA, USA).

### Cytokine Secretion

The cytokines interleukin-10 (IL-10), interferon-gamma (IFN-γ), and TNF-α were measured using a commercial ELISA kit (according to the guidelines established by BD Biosciences, San Diego, CA, USA). The cytokine levels present in the lung homogenates or cell culture supernatant were calculated based on a standard curve provided by the commercial kit.

### Cellular Migration

The percentages of migrating cells were determined at 72 h after Pb18 heat killed (HKPb18, 1x10^6^ cells) or thioglycolate (1.5 ml of 3% solution) inoculation into mice peritoneum. At four days post-inoculation, WT, and CD18^low^ mice were euthanized using 80 mg/kg of ketamine and 16 mg/kg of xylazine, and the peritoneal contents were washed with 5 ml of Hank’s solution for leukocyte collection. The total cell suspension was centrifuged, and the pellet was resuspended in RPMI-1640 with 5% of fetal bovine serum (Sigma-Aldrich, St. Louis, MO, USA). The cells were counted in a hemocytometer chamber in the presence of trypan blue. For differential counting, cytospins were stained with a Panótico^®^ kit (Laborclin, Brasília, DF, Brazil) to identify specific leukocytes (neutrophils, macrophages, and lymphocytes). The flow cytometry approach is used to analyze the pulmonary cell migration profile. Mice were infected with 1x105 heat-killed *P. brasiliensis* yeast (HKPb18) in the intranasal route. After three days, a collection of bronchoalveolar lavage (BAL) fluid was performed, and the mice were euthanized by CO2 overdose. Cold PBS and a 1-inch 22G catheter without a needle into the trachea were used to perform the lavage. Moreover, 5x105 cells were blocked with PBS, supplemented with 10% FBS for 1 h. After washing, cells were stained with anti-CD3 APC and anti-F4/80 FITC (Invitrogen, Carlsbad, Califórnia, USA) for 30 min, in ice, in the dark. Next, cells were washed two times and analyzed by Flow Cytometry.

### Ex-Vivo Phagocytosis Index

The kinetics of Pb18 internalization by the phagocytosis index was also investigated. At 6, 24, and 48 h after co-culture, the supernatant was removed, and the cells were stained with Panotico^®^ (Laborclin, Brasília, DF, Brazil). Phagocytosis was measured under the optic microscope (100×) in approximately one hundred cells. Phagocytosis index was determined by calculating the number of internalized cells in the phagocytosis and the yeasts’ average phagocytosed by the macrophages. A similar experiment was performed with fluorescent microscopy to identify and differentiate intra- and extracellular Pb18. Before co-incubation with macrophages, fungi were stained with 3 mg/ml of Fluorescein isothiocyanate (FITC) (Sigma-Aldrich, St. Louis, MO, USA) for 2 h in the dark at room temperature. Next, Pb18 was washed and incubated with macrophages for 24 h. After washing extracellular fungi, Calcofluor staining (Sigma-Aldrich, St. Louis, MO, USA) (10 ug/ml) was performed for 20 min at 37°C in the dark. After washing, phagocytosis index was measured by fluorescent microscopyA similar experiment was performed with fluorescent microscopy to identify and differentiate intra- and extracellular Pb18. Before co-incubation with macrophages, fungi were stained with 3 mg/ml of Fluorescein isothiocyanate (FITC) (Sigma-Aldrich, St. Louis, MO, USA) for 2 h in the dark at room temperature. Next, Pb18 was washed and incubated with macrophages for 24 h. After washing extracellular fungi, Calcofluor staining (Sigma-Aldrich, St. Louis, MO, USA) (10 ug/ml) was performed for 20 min at 37°C in the dark. After washing, phagocytosis index was measured by fluorescent microscopy. Data are expressed as mean ± SEM of three independent experiments.

### Statistical Analysis

Differences between the two experimental groups were analyzed by using ANOVA followed by the Bonferroni t-test. The p-value of <0.05 was considered significant

## Results

### β2 Integrin Influences Host Survival in *P. brasiliensis* Infection

**Figure 1:**
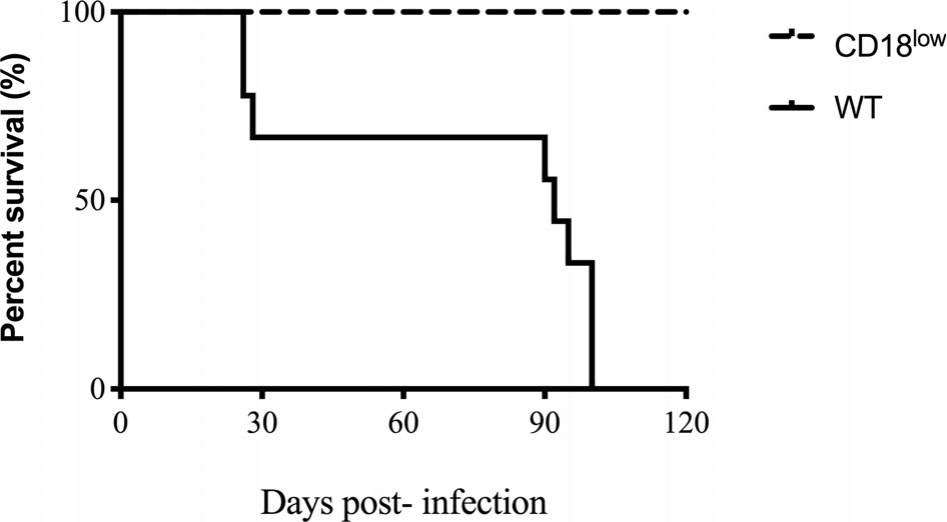
Survival curve of WT and CD18^low^. Mice were infected *via* i.v. with 10^6^ yeast forms of *P. brasiliensis* (Pb18) to mimic a chronic infection. Kaplan-Meier estimator was used to represent WT representative survival curves (solid line) and CD18^low^ (dashed line). For both groups, n=12. Data are expressed as the percentage of live animals observed for 120 days.

To evaluate the course of the chronic model of PCM in the β2 integrin low expression model, WT, and CD18^low^ mice were infected with a virulent strain of *P. brasiliensis* (Pb18) *via* i.v. route and monitored for survival for 120 days (**Figure 1**). Mice from both groups displayed clinical evidence of disease, and WT mice survived, on average, approximately 90 days. However, all infected CD18^low^ mice survived during the entire time course. At this point of infection, CD18^low^ mice were euthanized to perform the lung analysis, in which we observed lung granulomatous lesions and viable fungal cells within granuloma (data not shown). Nevertheless, CD18^low^ mice showed higher resistance to Pb18 infection in comparison to WT mice.

### Positive Outcome in CD18^low^ Mice After *P. brasiliensis* Infection

**Figure 2:**
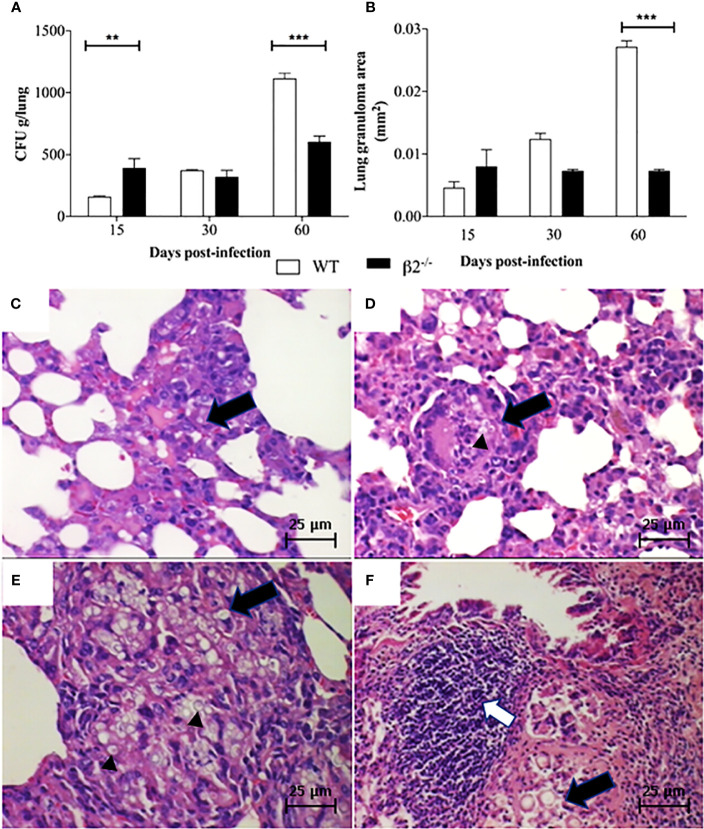
Fungal burden and granuloma in lung tissue. Mice were infected *via* i.v. with 10^6^ yeast forms of *P. brasiliensis* (Pb18) to mimic a disseminated infection. **(A)** Analysis of fungal burden of lung CFUs *in vivo*. **(B)** Measurement of the granuloma size in the lung tissue of infected animals. **(C, D)** Histological images of the granuloma formation (black arrows) and presence of yeast (black arrow head in Figure D) in WT and CD18^low^ mice at 15 days post-infection – 100x. **(E, F)** histological images represented granuloma formation (black arrows), and presence of yeast (black arrow head in Figure E) and lymphocytic infiltrate (white arrow) in WT and CD18^low^ mice 60 days post-infection – 100x. Data are presented as the mean ± SEM of three independent experiments (**significant difference p<0.01, ***significant difference p<0.001).

To investigate Pb infection kinetics in WT and CD18^low^ expression mice, lungs were aseptically removed and macerated to recover fungal burden. According to survive curve days related to mice mortality, the mice were euthanized at 15, 30, and 60 days post-infection. Sine *P. brasiliensis* is a facultative intracellular fungus and can grow outside macrophages, is option to use the whole tissue is to evaluate the total fungal population viability. A higher number of CFUs was observed in tissue recovered from CD18^low^ mice at 15 days post-infection when compared to WT animals. However, at 60 days post-infection, the CFU and the granuloma structures were higher in WT than CD18^low^ (**Figure 2A**). The fungal cells can also be observed in the granuloma (**Figures 2B–E**).

The histopathological analyses depict granuloma in both WT (**Figures 2B, C**) and CD18^low^ mice lung tissue (**Figures 2B, D**) at 15 days post-infection. WT mice lung granulomas were well organized, composed of epithelioid cells, lymphocytes, and a few multinuclear giant cells characterizing epithelioid granulomas. The CD18^low^ mice lungs showed granulomas with a reduction of the alveolar spaces, with lymphocyte infiltration and significant yeast levels at 15 days post-infection (**Figure 2D**). However, at 60 days post-infection, the granulomas in WT mice were less organized, showing an incipient pattern of granulomatous structures, with multinuclear giant cells within yeast forms fungus (**Figure 2E**). Besides, at 60 days post-infection, in the disease’s disseminated phase, the granulomas were more organized in CD18^low^ mice, with an increase in lymphocyte migration (**Figure 2F**). Comparing the granuloma lesion sizes between the groups, CD18^low^ mice granulomas impaired a lower area of lung (**Figure 2B**), so the lung was less compromised.

### Nitric Oxide and Cytokine Secretion Are Altered in the Course of CD18^low^ or WT Mice Infection

**Figure 3:**
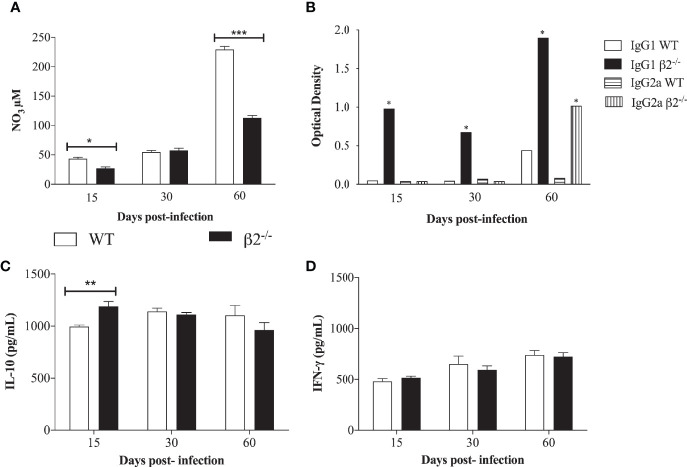
Quantification of nitric oxide, IgG1, and IgG2 and cytokine secretion in a *P. brasiliensis* infection systemic model. Mice were infected *via* i.v. with 10^6^ yeast forms of *P. brasiliensis* (Pb18) to mimic a chronic infection. **(A)** NO3 production was determined at 15-, 30-, and 60- days post-infection by Griess reagent. **(B)** IgG1 and IgG2a isotype levels in serum of WT and CD18^low^ mice were detected after 15-, 30- and 60- days post-infection by ELISA. The antibody titers were expressed in optic density (O.D). **(C, D)** IL-10 and IFN-γ secretion analyzed by ELISA from lung cell homogenates. Data are expressed as the mean ± SEM. (*Indicates significant difference p<0.05; **significant difference p<0.01, ***significant difference p<0.001).

The serums nitrate levels were measured in the WT and CD18^low^ *P. brasiliensis* infected mice to evaluate the nitric oxide production. At 15 and 60 days, post-infection, higher levels of nitrate were found in WT serum compared to CD18^low^ mice. At 60 days post-infection, the NO production was higher in WT mice (**Figure 3A**). No differences were observed at 30 days post-infection. Regarding the levels of IgG1 and IgG2a, antibody production was not observed in the serum of WT non-infected mice (data not shown). On the other hand, IgG1 levels were significantly higher in the serum of CD18^low^ infected mice compared with WT infected mice at all evaluated time points (**Figure 3B**). Nevertheless, levels of anti-*P. brasiliensis* IgG2a increased significantly in the serum of CD18^low^ mice only at day 60 post-infection.

Concerning cytokine secretion, IL-10, IL-12, TNF-α, and IFN-γ were quantified in lung cell homogenates. A significantly higher level of IL-10 in CD18^low^ mice was observed at 15 days post-infection in comparison to WT mice (**Figure 3C**). No significant differences were found when analyzing the presence of IFN-γ (**Figure 3D**). There were no statistical differences in TNF- α and IL-12 levels between the groups (**Supplementary Figure 1**). It is possible to correlate the higher CFU in CD18^low^ mice at 15 days post-infection with the low NO_3_ secretion at this time point. Among other functions, IL-10 is also stimulatory toward TCD8+ cells, and this can be associated with the decrease in the CFU at 60 days post-infection in CD18^low^ mice.

### Cellular Migration After *P. brasiliensis* Stimulus

**Figure 4:**
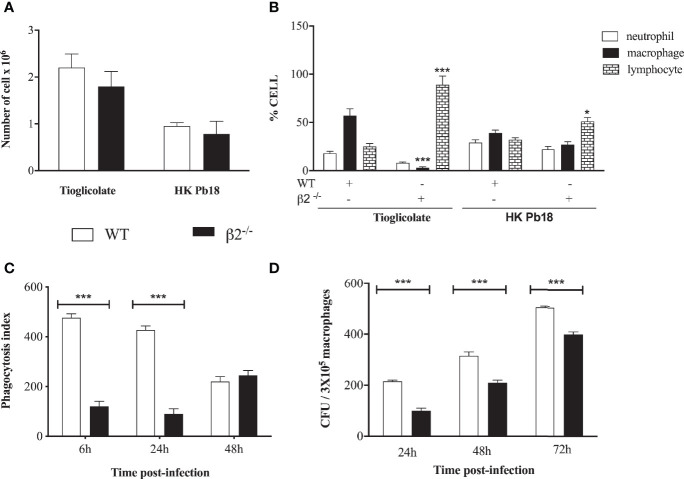
In vivo mobilization of cells in WT and CD18 low mice after i.p. inoculation of thioglycolate and heat-killed P. brasiliensis. **(A)** Total of cells migrating to the peritoneal cavity of WT and CD18^low^ mice at 4 days post-treatment with HKPb18 and thioglycolate. **(B)** Percentage of specific cell type counted in WT and CD18^low^ mice’s peritoneal cavity after treatment with HKPb18 and thioglycolate. **(C)** *In vitro* analysis of the phagocytosis index in WT and CD18^low^ mice derived macrophages at 6, 24, 48 h post-co-incubation with Pb18 (MOI 1:1). **(D)** Viable yeast recovered from WT and CD18^low^ macrophage-infected *in vitro* with Pb18 and plated at 24, 48, and 72 h post-co-incubation. Data are expressed as the mean ± SEM. (*Indicates significant difference p<0.05; ***significant difference p<0.001).

To analyze whether the differences between granuloma formation could be associated with cellular migration to the inflammatory site, since the molecules that depend on β2 integrin expression to promote the cell migration were decreased, we checked the leukocyte migration into the peritoneum after stimulation with heat-killed *P. brasiliensis* yeast (HKPb18) or thioglycolate. No difference in the percentage of total migrating leukocytes was detected between WT and CD18^low^ mice, although the thioglycolate treatment induced higher cellular migration (**Figure 4A**). Considering the differential migration into the WT peritoneum, thioglycolate stimulus caused more significant macrophage migration than neutrophils and lymphocytes (**Figure 4B**). The migration into the CD18^low^ mice peritoneum showed significantly higher lymphocyte migration levels after both treatments (**Figure 4B**). To compare the lung’s migration profile, we carried out the cell migration using heat-killed *P. brasiliensis* yeast (HKPb18) in the intranasal route (**Supplementary Figure 2**). The CD18low BAL confirmed the higher levels of lymphocyte migration to the tissue.

### The *In Vitro* Macrophage Activity

To evaluate the phagocytic activity of macrophages in WT and CD18^low^ mice, peritoneal macrophages from both groups of mice were co-cultivated *in vitro* with the yeast of Pb18. The fungal cells’ internalization and viability were assessed by phagocytic index, considering the colony-forming units (CFU) counting after 6 h, 24 h, 48 h, and 72 h of co-incubation.

It was found that macrophages from both WT and CD18^low^ mice were able to internalize Pb 18. Yeast phagocytosis increased more when the cells were opsonized with fresh serum compared to inactivated serum (data not shown). The phagocytosis index was significantly higher in WT macrophages than CD18^low^ cells at six and 24 h of co-culture (**Figure 4C**). There was a time-dependent increment in fungal burden in both macrophage groups during the kinetics. Considering the technical limitation to separate the adhered and internalized yeast, we carried out the phagocytosis index determination using fluorescent staining (**Supplementary Figure 3**). The results are similar to the **Figure 4C**, confirming a lower internalization of CD18^low^ macrophages. Nonetheless, we observed a higher number of viable *P. brasiliensis* recovered from WT when compared to CD18^low^ macrophages (**Figure 4D**).

**Figure 5:**
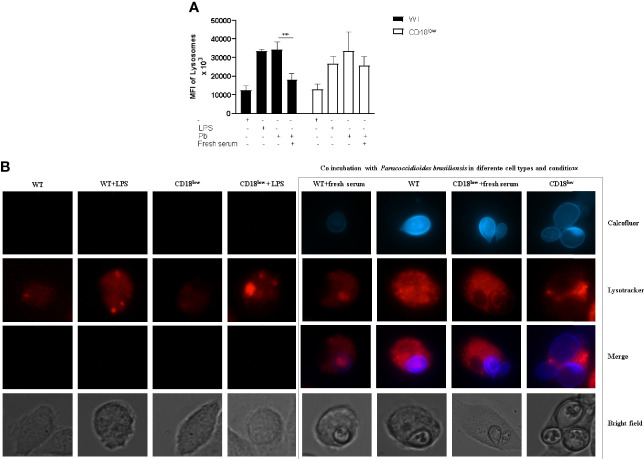
Lysosomal recruitment and acidification to evaluate macrophage activation. Detection of lysosomal acidification/recruitment in infected WT and CD18^low^ macrophages was performed using LysoTracker^®^ Red DND-99. **(A)** Representative pictures depicting calcofluor white stained fungi (blue) and Lysotracker staining (red). **(B)** Quantification of Mean Fluorescence Intensity (MFI) after Lysotracker staining of both WT and CD18^low^ macrophages after *P. brasiliensis* interactions *in vitro*. Images were taken using the Live Cell Imaging platform, and ImageJ analyzed MFI. Data are presented as mean ± SEM of at least three independent experiments (***indicates significant difference p<0.001).

Furthermore, macrophage activation through the acidification of the phagolysosome was also measured using Lysotracker staining and microscopy analysis (**Figure 5**). We also analyzed the mean fluorescence intensity (MFI) of lysosome staining. A lower intensity in the lysosome signal is observed in WT mice infected with fresh serum-opsonized *P. brasiliensis*. We did not observe differences in CD18^low^ macrophages’ lysosomal activities in relation to yeast opsonization.

## Discussion

The PCM outcome depends on several factors, among which fungal interaction with macrophages is a critical one. The interactions between resident fungi and macrophages determine the subsequent mechanisms of innate and adaptive immune activation. These processes are different when comparing the reactions of susceptible and resistant mice to experimental PCM (Pina et al., 2008). At the beginning of the infection, the susceptible mice developed better fungal growth control, with high NO and IL-12 production levels and increased expression of CD40, but with disseminated disease and low mice survival rate. On the other hand, the resistant mice showed a low production of NO, high levels of IL-10 and GM-CSF, and increased expression of Class II MHC molecules, with a well-controlled adaptive immune response. Other authors have suggested that the disease outcome is a consequence of initial pathogen recognition, followed by an exacerbated immune response associated with low fungal killing and CD4+ T-cells anergy (Pina et al., 2008). However, the juvenile form of PCM shows eosinophilia and high TGF-b levels and a T helper 2 cytokines patter (Mamoni et al., 2002), indicating that several cytokines are associated with the initial immune response, capable of modulating the disease outcomes.

There are some receptors associated with *P. brasiliensis* recognition, such as TLRs, CTLRs, and CRs. TLR-4 and TLR-2 are associated with a robust initial response and a non-controlled disease compared with deficient mice for these receptors (Calich et al., 2008; Loures et al., 2009; Loures et al., 2010). Contrarily, the Dectin-1 receptor is associated with increased response in resistant mice through inflammasome activation and IL-1β production (Feriotti et al., 2015). The role of a complement system has been previously described (Calich et al., 2008). However, the role of its receptors has not yet been fully elucidated. To verify the role of b2-integrin, we carried out *in vivo* experimental infections using CD18^low^ mice. The CD18^low^ Pb infected mice showed a higher fungal burden and lower NO_3_ levels at 15 days post-infection; however, the animal controlled the infection at 30 days post-infection. The resistance of these mice was confirmed by the survival curve, in which we detected a small number of CFUs in the lung compared to CFUs recovered from WT mice at 60 days post-infection. Our *in vivo* data corroborate reports on TLR-4 and TLR-2 KO infected mice (Pinto et al., 2006; Loures et al., 2009; Loures et al., 2010). It is possible to correlate the higher CFUs in CD18^low^ mice at 15 days post-infection with the low NO_3_ secretion at this time point. However, the levels of NO should also be analyzed in the lung. However, already at 15 dpi, we observed high levels of IL-10. Among other functions, IL-10 is also stimulatory towards TCD8+ cells, and this can be associated with the decrease in the CFUs at 60 days post-infection in CD18^low^ mice.

The histopathological and histocytometry analysis confirmed a progressive inflammatory response with extensive areas of granulomas in Pb infected mice. There are differences between granuloma structure between the two groups, and our results corroborate the granuloma formations described in susceptible (B10.A) and resistant (A/Sn) mice exposed to *P. brasiliensis* (Calich et al., 2008; Loures et al., 2009; Loures et al., 2010). To better understand if the smaller granulomatous lesions were associated with a low cellular migration to the lung, we carried out a migration assay. The lower expression of LFA-1 (CD11a/CD18) has no influence on total leukocyte migration. However, CD18^low^ mice showed more migration of lymphocytes than macrophages for both stimuli, explaining the increase of lymphocyte infiltration in the CD18^low^ lung. Another explanation for the higher levels of lymphocytes in the lung is that CD18 is also part of αDβ2, an adhesive and multiligand receptor, which is moderately expressed in circulating leukocytes, but it is upregulated in inflammatory macrophages. This adhesive property is microenvironment-dependent, and the b2-integrin density is important for migration to the inflammatory focus through extravascular space (Palecek et al., 1997). This receptor is also crucial to macrophage retention on tissue, promoting chronic inflammation (Yakubenko et al., 2008). Our results showed significantly lower macrophage migration into the peritoneum after thioglycolate stimulus, which can be explained by a low expression of αDβ2. The microenvironment with macrophage depletion and a rise in lymphocytes can modulate granuloma formation, fungal viability, and resistance to the infection, as observed in our results.

Furthermore, the granulomas can be modulated by NO production. The treatment of susceptible mice with an inhibitor of NOS2 (inducible nitric oxide synthase 2) induces a considerable increase in the number and the size of liver granulomas. Simultaneously, in animals with the regular expression of NO, there were smaller granulomas, despite the worse immunological parameters during infection. These results suggest that NO levels are closely correlated with the extension of granulomatous lesions. The cessation of NO production during the initial phase may cause more severe disease. In contrast, the overproduction of this mediator is associated with susceptibility (Yakubenko et al., 2008).

In our work, the NO_3_ serum levels of CD18^low^ mice in response to *P. brasiliensis* infection were higher only at 15 days post-infection. However, this production did not increase during infection progression, as observed in WT mice levels. The correlation between the activation of NO production and fungal lung infections and immunosuppressant mechanisms has been previously reported during a *P. brasiliensis* mouse infection. These studies revealed that, although NO is an essential microbicidal mechanism for macrophages infected with *P. brasiliensis*, the overproduction of NO in PCM contributes to immunosuppression during the disease (Nascimento et al., 2002; Yakubenko et al., 2008). One of the NO immunosuppressive pathways reduces class II MHC expression, with antigen-presentation for T-cells, decreased IFN-γ levels, and inhibition of IFN-γ-dependent production of NO synthase, which prevents excessive NO formation and tissue injury (Sicher et al., 1994; Bocca et al., 1998; Bocca et al., 1999). The physiological role of NO in host immunity suppression to avoid tissue injury can also modulate granuloma formation. In some cases, it can increase host susceptibility, as described in the PCM model, and confirmed in this work.

In several infection models, including PCM, macrophages’ fungicidal activity against fungi has been associated with IFN−γ-macrophage activation and, consequently, NO production induced by NO synthase. Therefore, NO induction depends on the synergy between Th1 cytokines, TNF-α and IFN-γ, and/or cellular constituents of the pathogen (Brummer et al., 1988; Xie et al., 1991; Bocca et al., 1998; Bogdan et al., 2000). The phagocyte’s fungicidal activity seems to be dependent on the equilibrium between stimulating and suppressing cytokines during interaction with the fungus to regulate the NO levels. The early production of IL-10 by CD18^low^ mice is probably associated with a reduction in NO production at a later time point and the prevention of its immunosuppressive role.

Th1 lymphocytes are essential for an effective cellular immune response against intracellular pathogens because of IFN-γ secretion, which activates macrophages and stimulates T CD8^+^ cells. In murine models, IFN-γ also induces the production of both IgG2a and IgG3, which contribute to antimicrobial immunity through their complementary and opsonization activities. Our data revealed that CD18^low^ mice underwent a significant increase in IgG1 produced during the time analyzed and in IgG2a levels at 60 days post-infection compared to WT mice. The phagocytosis efficacy of *Cryptococcus neoformans* by macrophages is mediated by opsonization of IgM and IgA antibodies and is correlated with CR3 expression (Taborda and Casadevall, 2002). However, it was demonstrated that when blocking CR3, the phagocytosis mediated by IgG1 was partially inhibited, suggesting that phagocytosis mediated by IgG1 is not entirely dependent on β integrins and that CD18^low^ macrophages used a similar pathway to internalize the yeast cells. The pattern of IgG production in CD18^low^ mice in experimental PCM needs to be better explored; however, we can presume that the levels of IgG1 correlate with a non-inflammatory response at the beginning of the infection and the IgG2a with a Th1 response at the end of the infection.

Macrophages can play different roles in the lung, as shown in pulmonary infection caused by *C. neoforman*s or *A. fumigatus*. The populations associated with tolerance and tissue homeostasis have produced low levels of CXCL2 and high levels of IL-10 and complement component 1q (C1q) (Xu-Vanpala et al., 2020). Activation of the complement system by *P. brasiliensis*, either by the classical or alternative pathways, results in yeast cell opsonization, promoting phagocytosis and fungicidal killing (Calich et al., 2008). We analyzed the kinetics of the *in vitro* phagocytosis of *P. brasiliensis* yeast opsonized with fresh serum by macrophages from WT and CD18^low^ mice, and WT macrophages internalized more yeast cells.

Several works have demonstrated that the phagocytosis of microorganisms such as *C. neoformans* (Kelly et al., 2005; Luo et al., 2006), *C. albicans* (Triebel et al., 2003; Gruber et al., 1998), *M. tuberculosis* (Velasco-Velasquez et al., 2003), and *L. monocytogenes* (Drevets et al., 1996) in the presence of fresh human serum is more efficient than in the presence of inactivated serum. The low phagocytosis rate of yeasts by CD18^low^ macrophages emphasizes the importance of complement receptors in internalization. Nevertheless, the phagocytosis of *P. brasiliensis* yeast cells can occur by other receptors, which may explain why the phagocytic activity increased progressively after 24 h, although at 48 h post-infection, we observed no differences in the phagocytosis index between the groups. The fungal load in macrophages increased during the kinetic analyses for both groups; however, despite showing the same number of internalized yeast cells at 48 h, we observed a reduction in the viability of Pb in CD18^low^ macrophages. These results are consistent with more efficient macrophage killing of fungal cells in this group. The complement receptors (CR1, CR3, and CR4) expressed in macrophages are important for recognition and adhesion of *M. tuberculosis*. As observed in our results, similar studies using *M. tuberculosis* have demonstrated that bacteria also exploit receptors to enter macrophages’ cytoplasm (Hirsch et al., 1994; Velasco-Velasquez et al., 2003) and modulate phagocyte activation and disease outcome.

The ability of microorganisms to be destroyed has been related to phagocyte intracellular acidification. When this mechanism fails, improper phagosome acidification is positively associated with the intracellular survival of *C. neoformans* and *Leishmania* (Hirsch et al., 1994; Xu-Vanpala et al., 2020). The β2 integrin receptor has been well described using a *Leishmania* model, corroborating several studies that have associated the presence of CR3 with a delay in lysosome recruitment and phagolysosome maturation (Carter et al., 2009; Polando et al., 2013; Ricardo-Carter et al., 2013). Our results showed increased lysosomal recruitment on both WT and β2-/- infected macrophages with non-opsonized fungal cells. The opsonization of yeast cells with fresh serum produced reduced lysosome recruitment in WT macrophages, suggesting low phagocyte activation. β2 integrin is one of two chains in CR3 conformation, and other components can be related to the lysosomal recruitment pathway in *P. brasiliensis* infection, such as CR1, related to early phagolysosome maturation.

Nevertheless, the relationship between CD18 and PCM in an early phase of infection must be evaluated. Regarding the role of CR3 in diverse species of intracellular pathogens, this receptor can crosstalk with TLRs and interfere in several diseases’ pathogenesis. The role of CR3/TLR2 or CR3/TLR4 has not been studied in PCM, and the understanding of *P. brasiliensis* survival in macrophages should be better explored.

In the present manuscript, we focused on active antifungal macrophages after infections with *P. brasiliensis.* Our data suggest that the presence of β2 integrin is associated with an initial inflammatory response and intracellular fungal survival. The beta chain protein could serve as a “safe passage” for the fungus, supporting its proliferation, and its default would lead the fungus to enter the macrophages *via* a less “friendly” receptor, able to activate this phagocytic cell more efficiently.

## Data Availability Statement

The raw data supporting the conclusions of this article will be made available by the authors, without undue reservation.

## Ethics Statement

The animal study was reviewed and approved by The Animal Ethics Committee of then University of Brasilia (UnBDOC n°. 33798/2007).

## Author Contributions

AB and AA- conceived, and designed research. SM, JN, EC, PHB, and AS - conducted experiments. AB, AT, LM, and LF - contributed with reagents and analytical tools. SM, JN, FC, PHB, and AR - analyzed data. AB, SM, AA, and LM - wrote the manuscript. All authors contributed to the article and approved the submitted version.

## Funding

We would like to thank Dr. Magda Verçosa Carvalho Branco from UNICEUB for providing isogenic C57Bl/6 mice. This research was funded by a grant from CNPq (Conselho Nacional de Pesquisa - 306515/2019-9), FAPDF (Fundação de apoio à Pesquisa do Distrito Federal –193.000496/2009 and 193.000417/2016), and CAPES.

## Conflict of Interest

The authors declare that the research was conducted in the absence of any commercial or financial relationships that could be construed as a potential conflict of interest.

## Supplementary Material

Supplementary Figure 1 Quantification TNF-α and IL-12 secretion in a *P. brasiliensis* infection systemic model. Mice were infected *via* i.v. with 10^6^ yeast forms of *P. brasiliensis* (Pb18) to mimic a chronic infection. **(A)** TNF-α and **(B)** IL-12 secretion analyzed by ELISA from lung cell homogenates. Data are expressed as the mean ± SEM. (* Indicates significant difference p<0.05; **significant difference p<0.01, *** significant difference p<0.001).

Supplementary Figure 2 Cell migration in BAL of WT and KO animals. Wild Type and CD18 low mice were infected with heat killed Pb18 in the intranasal route. After euthanasia, BAL cells were collected and stained with anti CD3 and anti- F4/80 before Flow Cytometry analyses. **(A)** Dot plot of WT cells depicting lymphocytes and macrophages after specific staining. **(B)** Dot plot of CD18low cells depicting lymphocytes and macrophages after specific staining. **(C)** Quantification of anti-CD3 and anti-F4/80 fluorescence in both cell populations of WT and CD18low BAL. Data are expressed as the mean ± SEM. (*Indicates significant difference p<0.05; **significant difference p<0.01, ***significant difference p<0.001).

Supplementary Figure 3 Phagocytosis index of Pb18 by WT and CD18low cells. Pb18 was stained with Fluorescein isothiocyanate (FITC) before co-incubation with macrophages. Next, extracellular fungi were stained with Calcofluor. Phagocytosis index was analyzed by fluorescent microscopy. **(A)** Picture panel depicting intracellular Pb18 (green) and extracellular Pb18 (blue). **(B)** Quantification of phagocytosis index of both WT and KO cells. Data are expressed as the mean ± SEM. (*Indicates significant difference p<0.05; **significant difference p<0.01, ***significant difference p<0.001).
