# Supplementary figures and images for: β2 Integrin-Mediated Susceptibility to Paracoccidioides brasiliensis Experimental Infection in Mice
Source: Front Cell Infect Microbiol. 2021 Mar 16;11:622899. doi: 10.3389/fcimb.2021.622899 (PMC8007971; doi:10.3389/fcimb.2021.622899)

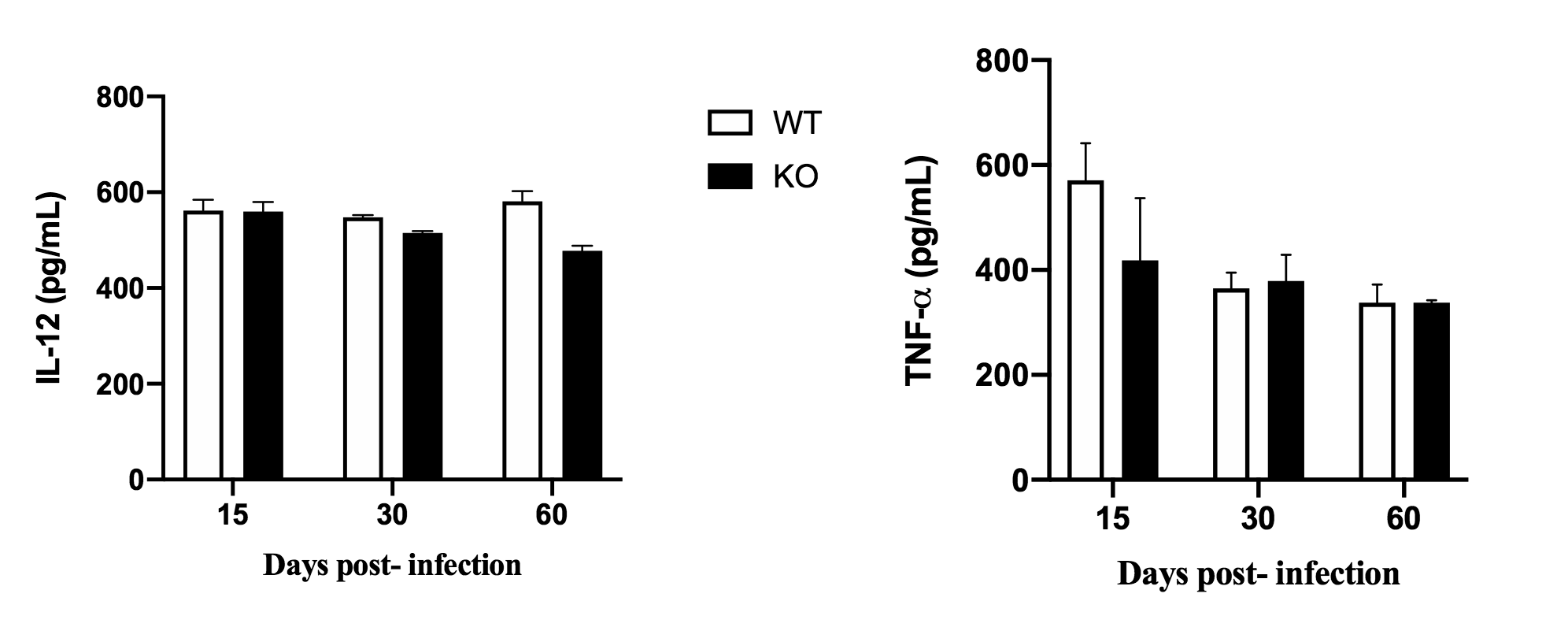

Supplement: Supplementary Figure 1 — Quantification TNF-α and IL-12 secretion in a P. brasiliensis infection systemic model. Mice were infected via i.v. with 106 yeast forms of P. brasiliensis (Pb18) to mimic a chronic infection. (A) TNF-α and (B) IL-12 secretion analyzed by ELISA from lung cell homogenates. Data are expressed as the mean ± SEM. (* Indicates significant difference p<0.05; **significant difference p<0.01, *** significant difference p<0.001). [file Image_1.tiff]

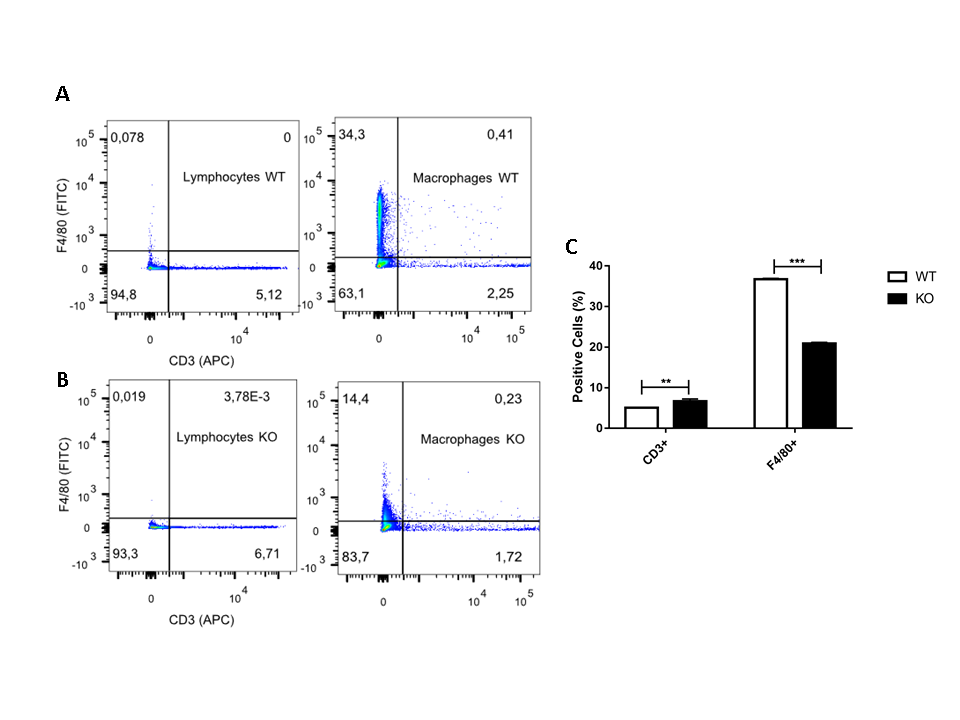

Supplement: Supplementary Figure 2 — Cell migration in BAL of WT and KO animals. Wild Type and CD18 low mice were infected with heat killed Pb18 in the intranasal route. After euthanasia, BAL cells were collected and stained with anti CD3 and anti- F4/80 before Flow Cytometry analyses. (A) Dot plot of WT cells depicting lymphocytes and macrophages after specific staining. (B) Dot plot of CD18low cells depicting lymphocytes and macrophages after specific staining. (C) Quantification of anti-CD3 and anti-F4/80 fluorescence in both cell populations of WT and CD18low BAL. Data are expressed as the mean ± SEM. (*Indicates significant difference p<0.05; **significant difference p<0.01, ***significant difference p<0.001). [file Image_2.tif]

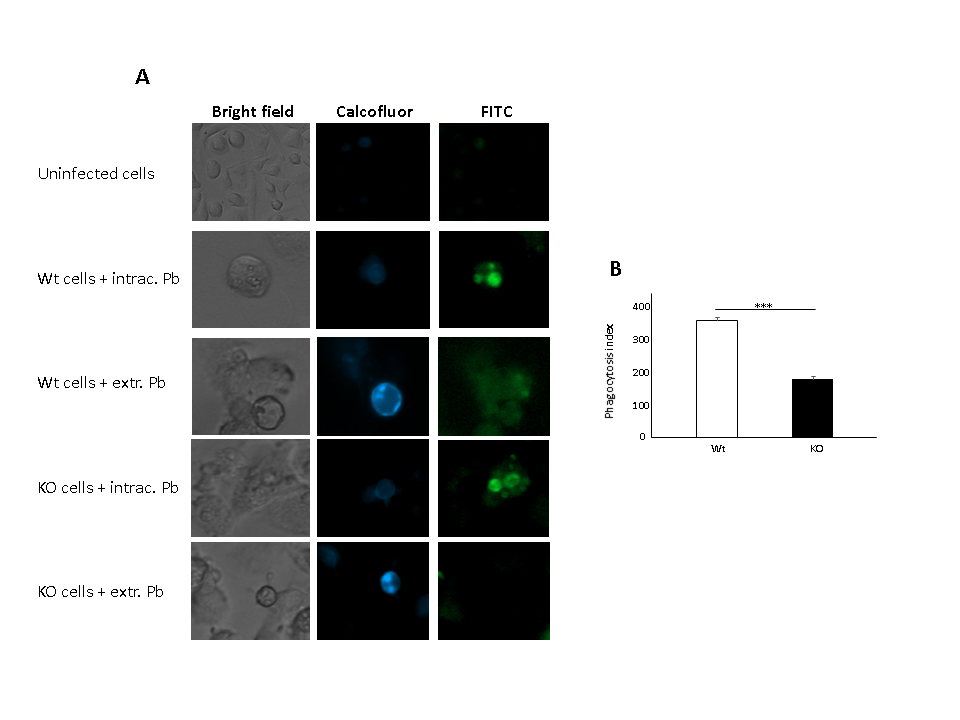

Supplement: Supplementary Figure 3 — Phagocytosis index of Pb18 by WT and CD18low cells. Pb18 was stained with Fluorescein isothiocyanate (FITC) before co-incubation with macrophages. Next, extracellular fungi were stained with Calcofluor. Phagocytosis index was analyzed by fluorescent microscopy. (A) Picture panel depicting intracellular Pb18 (green) and extracellular Pb18 (blue). (B) Quantification of phagocytosis index of both WT and KO cells. Data are expressed as the mean ± SEM. (*Indicates significant difference p<0.05; **significant difference p<0.01, ***significant difference p<0.001). [file Image_3.tif]
